# Effect of the Active Cycle of Breathing Technique on Perioperative Outcome in Individuals With Esophagectomy: A Quasi-Experimental Study

**DOI:** 10.3389/fsurg.2021.735947

**Published:** 2021-10-15

**Authors:** Si-Wen Zhang, Lei-Lei Wu, Hong Yang, Chuan-Zhen Li, Wei-Jin Wei, Min Wang, Guo-Wei Ma, Jiu-Di Zhong

**Affiliations:** ^1^The Department of Thoracic Surgery, Sun Yat-sen University Cancer Center, State Key Laboratory of Oncology in South China, Collaborative Innovation Center for Cancer Medicine, Guangzhou, China; ^2^Department of Thoracic Surgery, Shanghai Pulmonary Hospital, School of Medicine, Tongji University, Shanghai, China; ^3^Guangdong Esophageal Cancer Institute, Guangzhou, China

**Keywords:** active cycle of breathing technique, perioperative outcome, esophageal carcinoma, anastomotic leakage, esophagectomy

## Abstract

**Background:** The effect of active cycle of breathing technique (ACBT) on EC patients has not been well elucidated. In this research, we aim to explore the effect of ACBT on the perioperative outcomes in patients with esophageal carcinoma who underwent esophagectomy.

**Methods:** Patients who underwent esophagectomy in an academic institution from December 2017 to July 2019 were included in this study. In a quasi-experimental study, participants were randomly divided into an experimental group (active cycle of breathing technique, *n* = 107) and an observational group (*n* = 106) by drawing lots. The chi-squared test, Cochran–Mantel–Haenszel test, Logistic regression analysis, and Kruskal–Wallis test were used to analyze data. A two-sided *P* value <0.05 was considered statistically significant. The primary observational endpoint was the mean weight of the sputum. Other outcomes included the six-min-walk test (6MWT), Borg scale, anastomotic leakage, and the length of hospital stay.

**Results:** 95 patients underwent minimally invasive surgery, and 118 patients received open surgery. There were 16 patients with anastomotic leakage in the present study, and we found that patients in the observational group had higher odds of anastomotic leakage. The results showed that the mean weight of the sputum in the observation group was lighter than that of the experimental group. After esophagectomy, the experimental group had better outcomes than the observation group (Borg scale: 2.448 vs. 1.547; 6-MWT: 372.811 vs. 425.355m, all *P* < 0.05). The mean length of hospital stay was longer in the observation group (17.953 days) than that in the experimental group (12.037 days, *P* = 0.01). We also found that the observational group had a higher discharge ratio over 2 weeks in all cohort (adjusted OR 2.487, 95% confidence intervals 1.147–5.392, *P* = 0.021).

**Conclusion:** Active cycle of breathing technique may improve the perioperative outcomes and decrease the length of hospital stay after surgery in patients with esophageal cancer. However, we need more researches to validate these findings.

## Introduction

Esophageal carcinoma (EC) is one of the most common malignancies worldwide, ranking seventh and sixth in terms of incidence and mortality in the world cancer spectrum, respectively (1). The major histological subtypes of EC include squamous cell carcinoma and adenocarcinoma, with esophageal squamous cell carcinoma accounting for most cases (1–3). At present, the treatment of esophageal cancer mainly includes surgery, radiotherapy, chemotherapy, and immunotherapy, and surgery is the main treatment. Despite improvements in minimally invasive treatment strategies (4, 5), surgery remains the mainstream curative management. However, esophagectomy is a complex procedure that has a high rate of postoperative mortality and morbidity, including pulmonary complications (6–10). Because of complications, the length of hospital stay is significantly long. The duration of recovery in the perioperative period was consistent in patients with EC. In this period, patients always experience fatigue and pain (11).

The weight of sputum is considered a predictive indicator of pulmonary complications (12). Several studies have indicated that sputum contains microorganisms that could cause diseases, such as pneumonia and systemic inflammatory response syndrome (7, 13). The Borg scale was used to assess the tolerance of patients who engage in aerobic exercises (14, 15). The 6-min walk test (6-MWT) can be used to identify heart and lung function to some extent after surgery (16–18). Moreover, the Borg scale and 6-MWT can be utilized to evaluate the recovery of patients after surgery.

In the beginning, the active cycle of breathing technique (ACBT) provides a short-term improvement in secretion clearance in individuals with lung diseases, particularly non-cystic fibrosis bronchiectasis and cystic fibrosis bronchiectasis (19–24). In patients with cystic fibrosis, additional physical treatment, including ACBT, can improve muscle function, oxygen saturation, and small airway function and can reduce dyspnea. ACBT, which comprised breathing control and huff, was developed using the forced expiration technique and resulted in fast-track recovery after thoracic surgery. A typical ACBT is composed of breathing control, thoracic expansion exercises, and the forced expiration technique. The frequency of ACBT is flexible. However, all parts of the cycle must be included and interspersed with breathing control. A previous study has indicated that ACBT could increase the volume of sputum and the 6-MWT and Borg scale scores in patients with lung cancer who underwent lung resection (25).

The current study focused on the effect of ACBT on the perioperative outcomes in patients with EC who underwent esophagectomy. The following clinical observational indicators were used to assess the effect of ACBT: the weight of sputum, length of hospital stay, Borg scale score, anastomotic leakage, and 6-MWT score.

## Materials and Methods

### Study Cohort

**Figure 1:**
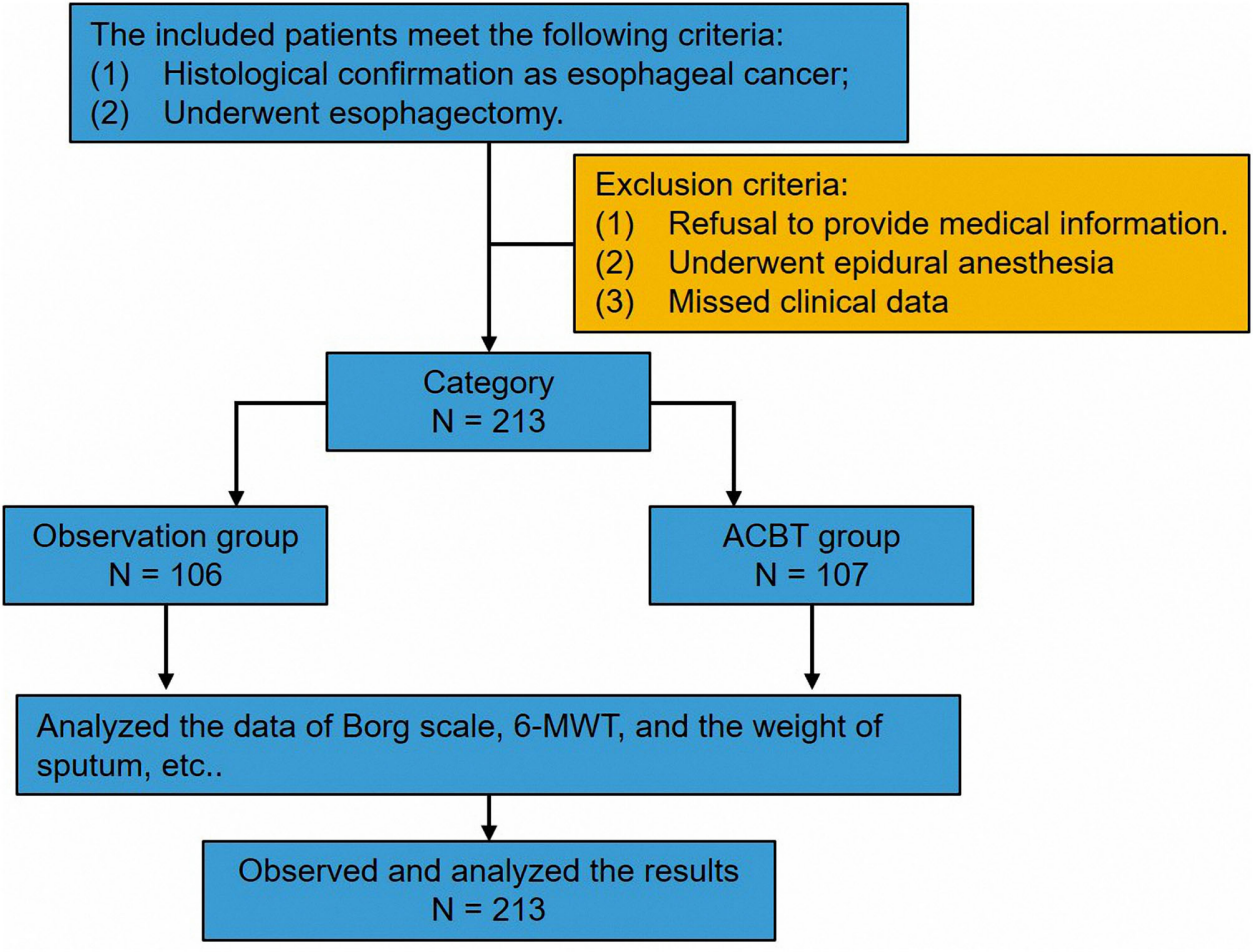
The follow chart of this research.

This study was approved by the ethics committee of Sun Yat-sen University Cancer Center (No. GYX2017-003). All patients signed the informed consent form. The study cohort comprised 213 patients from the Department of Thoracic Surgery in our hospital. Patients who were diagnosed of EC via histopathologic examination and underwent esophagectomy from December 2017 to July 2019 were recruited. Those patients did not receive neo-adjuvant therapy. In a quasi-experimental study, participants were randomly divided into an experimental group (active cycle of breathing technique, *n* = 107) and an observational group (*n* = 106) by drawing lots. In the cohort, predicted postoperative forced expiratory volume in 1 s of all patients was higher than 70%. The process of screening the patients is shown in Figure 1. All patient records were anonymized before analyses. We included information about sex, age, surgical approach, pTNM stage, histological type, tumor location, the weight of sputum, the length of hospital stay, smoking index (the number of cigarettes a person smokes per day multiplied by the number of years of smoking), Borg scale score, and 6-MWT score.

### ACBT, 6-MWT Score, Borg Scale Score, and Weight of Sputum

**Figure 2:**
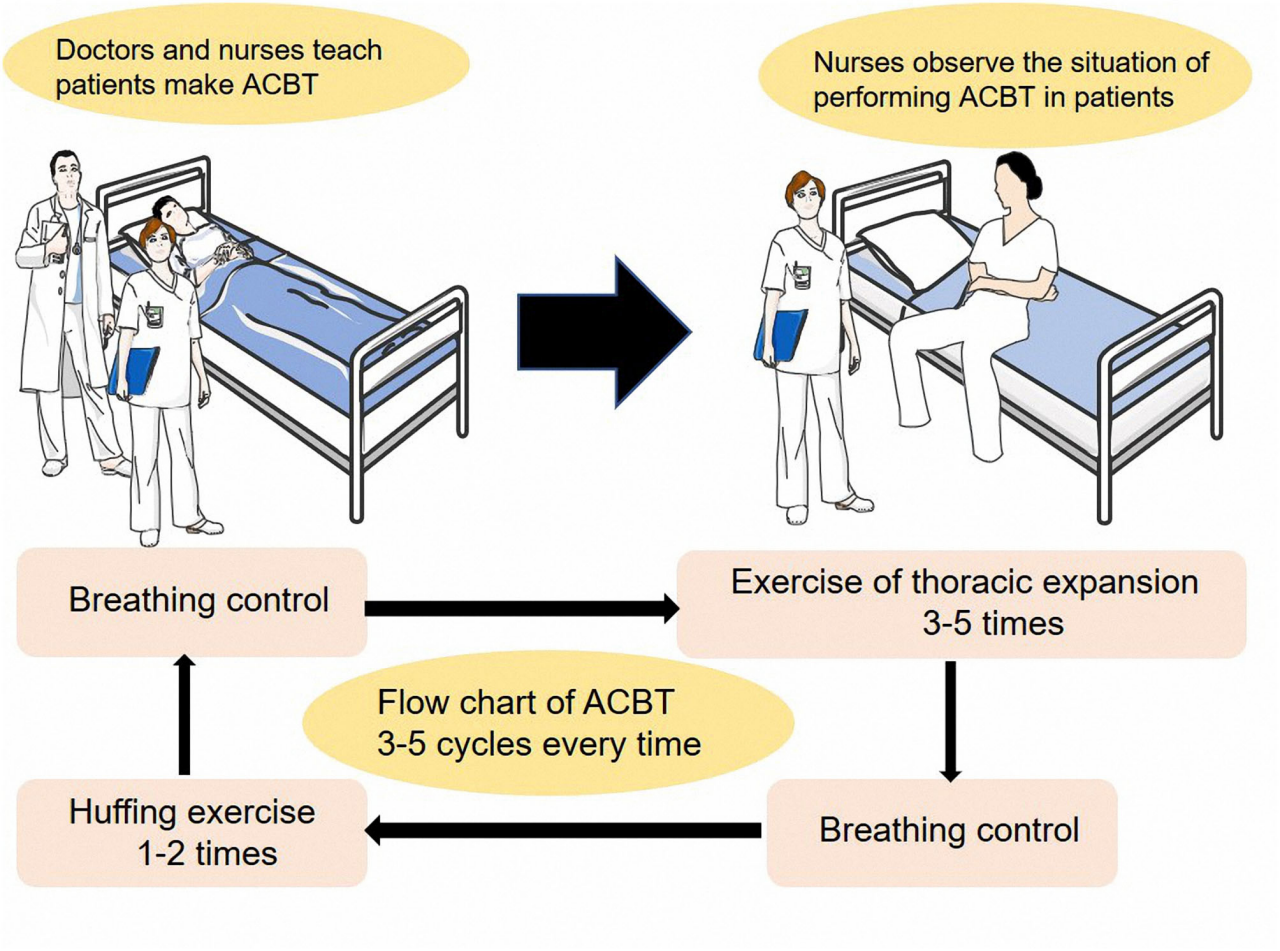
The sketch map of ACBT.

The patients in the observation group engaged in routine perioperative breathing exercises, including cough exercises and deep breathing. Before surgery, the patients in the observation group learned routine breathing exercises, and after surgery, they executed the coughing exercise three to five times per day, according to the postoperative rehabilitation instruction, with the help and guidance of a nurse. The patients in the ACBT group received ACBT training in addition to routine perioperative training. ACBT included thoracic expansion exercises, breathing control, and the forced expiration technique, interspersed with breathing control. The patients were instructed to repeat the cycle three to five times and even more if the treatment was tolerable, and each session lasted for 15–20 min. The detailed approach was based on the study of Mei Yang (25). The patients assumed a relaxed seating or reclined position before starting (Figure 2).

The 6-MWT was performed before and 10 days after surgery by a registered nurse. The patients walked for 6 min on a closed, ruled corridor. All patients were informed of the purpose, method, and results of the study using the 6-MWT. According to previous research approaches, the patients were allowed to set the walking speed and to stop if needed, and they were instructed to walk as far as they could.

The Borg scale is a 10-point scale, where sitting is assigned a score of 0, moderate-intensity activity 5 or 6, and all-out effort 10. These items can identify obvious increases in heart rate and breathing rate. Using the same scale, vigorous-intensity activity is given a score of 7 or 8, which indicates significant increases in heart rate and breathing rate. The patients were evaluated using the Borg scale before and after surgery. To assess heart and lung function using the Borg scale, we used the 6-MWT as an aerobic exercise.

We collected sputum samples before and on the first, second, and third days after surgery in a clean sterile pot, and these samples were then weighed (New Health electronic balance, model 20161206, manufactured in China in 2016; accurate to 0.01 g).

### Surgery

Surgeries were performed according to the following standard surgical approaches: Sweet, McKeown, and Ivor Lewis procedures. The study cohort all underwent thoracoabdominal lymphadenectomy.

### Histological Type

The patients exhibited the following histological types: adenocarcinoma, squamous cell carcinoma, adenosquamous carcinoma, small-cell carcinoma, and melanoma.

### Statistical Analyses

Statistical analyses were performed using the Statistical Package for the Social Sciences software version 25.0 (IBM SPSS, Inc., Chicago, IL, USA) and GraphPad Prism 8.0 (https://www.graphpad.com/scientific-software/prism/). The correlations between groups and clinicopathological characteristics were assessed using the chi-squared test, Logistic regression analysis, and Cochran–Mantel–Haenszel (CMH) test. The Kolmogorov–Smirnov test was used to assess the normality of the data recorded. Normally distributed data were expressed as mean (standard deviation, SD) values, and between-group differences were analyzed using the student's *t*-test. Meanwhile, non-normally distributed data were analyzed using the Kruskal–Wallis test. A two-sided *P* value <0.05 was considered statistically significant.

## Results

### Characteristics of the Patients

In this group of patients, 12 of them had adenocarcinoma, 3 patients had adenosquamous carcinoma, 1 patient had melanoma, 1 patient had small cell carcinoma, and the rest of the patients had squamous carcinoma. The clinical characteristics of the patients in the observation and ACBT groups are depicted in Table 1. In total, 125 (58.7%) and 72 (33.8%) patients underwent the McKeown and Ivor Lewis procedures, respectively. Most patients (*n* = 118, 55.4%) underwent open surgery, and 95 (44.6%), minimally invasive surgery. There were 16 patients with anastomotic leakage in the present study including 1 case in the ACBT group and 15 patients in the observation group. Before the specific statistical analysis, we assessed continuous numerical data using the Kolmogorov–Smirnov test. The results of the normality test are presented in Table 2. In both groups, no significant differences were observed in terms of gender, the pathway of surgery, tumor location, pTNM stage, surgical approach, type of surgery, age, and smoking index (Table 1, all *P* > 0.05). Before surgery, the following indicators did not significantly differ in both groups: the weight of sputum, Borg scale score, and 6-MWT score (Table 1).

**Table 1:** The associations of clinicopathological characteristics in the groups of observation and ACBT.

	**All (*N* = 213)**	**Observation (*N* = 106)**	**ACBT (*N* = 107)**	
**Variables**	**No. of patients(%)/ Mean (± SD)**			***P*** **value**
**Sex**				0.590^**^
Male	168 (78.9%)	82 (77.1%)	86 (80.4%)	
Female	45 (21.1%)	24 (22.9%)	21 (19.6%)	
**Pathway of surgery**				0.329^**^
Left thorax	33 (15.5%)	19 (17.9%)	14 (13.1%)	
Right thorax	180 (84.5%)	87 (82.1%)	93 (86.9%)	
**Tumor location**				0.108^*^
Upper thoracic	22 (10.3%)	13 (12.4%)	9 (8.4%)	
Middle thoracic	110 (51.6%)	61 (57.1%)	49 (45.8%)	
Lower thoracic	78 (36.6%)	31 (29.5%)	47 (43.9%)	
Gastroesophageal junction	3 (1.4%)	1 (1.0%)	2 (1.9%)	
**TNM stage**				0.825^*^
Tis	9 (4.2%)	4 (3.8%)	5 (4.7%)	
I	42 (19.7%)	18 (17.0%)	24 (22.4%)	
II	78 (36.6%)	40 (37.7%)	38 (35.5%)	
III	79 (37.1%)	42 (39.6%)	37 (34.6%)	
IV	5 (2.3%)	2 (1.9%)	3 (2.8%)	
**Approach of surgery**				0.953^**^
Sweet	16 (7.5%)	8 (7.6%)	8 (7.5%)	
Ivor-Lewis	72 (33.8%)	37 (34.9%)	35 (32.7%)	
McKeowm	125 (58.7%)	61 (57.5%)	64 (59.8%)	
**Types of surgery**				0.892^**^
Minimally invasive	85 (39.9%)	43 (40.6%)	42 (39.3%)	
Open	118 (55.4%)	59 (55.7%)	59 (55.1%)	
Robot-assisted	10 (4.7%)	4 (3.7%)	6 (5.6%)	
**Anastomotic leakage**				0.001^*^
No	199 (93.4%)	93 (87.7%)	106 (99.1%)	
Yes	14 (6.6%)	13 (12.3%)	1 (0.9%)	
**The weight of sputum (g)**				
Before surgery	2.255 ± 0.412	2.289 ± 0.433	2.220 ± 0.389	0.206^***^
1 day after surgery	7.759 ± 1.916	6.676 ± 1.772	8.831 ± 1.379	<0.001^***^
2 days after surgery	13.934 ± 3.726	11.241 ± 2.359	16.602 ± 2.795	<0.001^***^
3 days after surgery	18.946 ± 6.112	13.917 ± 3.405	23.928 ± 3.592	<0.001^****^
**Smoking index**	441.596 ± 464.54	435.472 ± 471.67	447.664 ± 459.51	0.722^***^
**Age (year)**	60.380 ± 8.334	60.410 ± 8.238	60.360 ± 8.467	0.853^***^
**Borg scale**				
Before surgery	0.347 ± 0.399	0.344 ± 0.399	0.351 ± 0.402	0.918^***^
After surgery	1.995 ± 0.788	2.448 ± 0.601	1.547 ± 0.689	<0.001^***^
**6-MWT**				
Before surgery	541.140 ± 57.96	536.200 ± 57.69	546.040 ± 58.08	0.024^***^
After surgery	399.207 ± 66.49	372.811 ± 63.35	425.355 ± 56.77	<0.001^***^
**Time of postoperative hospital stay (day)**	14.981 ± 15.84	17.953 ± 18.961	12.037 ± 11.321	0.010^***^
**Time to removal of thoracic tube (day)**	8.66 ± 8.50	9.81 ± 10.70	7.44 ± 4.95	0.330^***^
**Time to removal of gastric tube (day)**	10.64 ± 14.27	13.73 ± 19.02	7.66 ± 5.78	0.057^***^
**Time to removal of nutrition tube (day)**	11.94 ± 22.38	13.68 ± 20.28	10.18 ± 24.33	0.107^***^
**Over morbidity**				<0.001^*^
Pulmonary infection	17 (30.4%)	14 (32.6%)	3 (23.1%)	
Pulmonary atelectasis	8 (14.3%)	6 (14.0%)	2 (15.4%)	
Pneumothorax	14 (25.0%)	10 (23.2%)	4 (30.8%)	
Pleural effusion	11 (19.6%)	8 (18.6%)	3 (23.0%)	
Respiratory failure	6 (10.7%)	5 (11.6%)	1 (7.7%)	

*ACBT, active cycle of breathing technique; 6-MWT, 6-min walk test*;

* *Fisher's exact test*;

** *Chi-squared test*;

*** *Kruskal–Wallis test*;

**** *Cochran–Mantel–Haenszel test*.

**Table 2:** Normality test of each variable.

**Variables**	**Kolmogorov-Smirnov**		
	***Statistics***	***df***	***P value***
Weight of sputum (before surgery)	0.128	213	<0.001
Weight of sputum (1 day after surgery)	0.095	213	<0.001
Weight of sputum (2 days after surgery)	0.076	213	0.005
Weight of sputum (3 days after surgery)	0.146	213	<0.001
Borg scale (before surgery)	0.324	213	<0.001
Borg scale (after surgery)	0.272	213	<0.001
6-minutes-walk test (before surgery)	0.143	213	<0.001
6-minutes-walk test (after surgery)	0.107	213	<0.001
Time of postoperative hospital stay (day)	0.304	213	<0.001
Time to removal of thoracic tube (day)	0.232	213	<0.001
Time to removal of gastric tube (day)	0.311	213	<0.001
Time to removal of nutrition tube (day)	0.297	213	<0.001

### Weight of Sputum, 6-MWT Score, and Borg Scale Score

**Figure 3:**
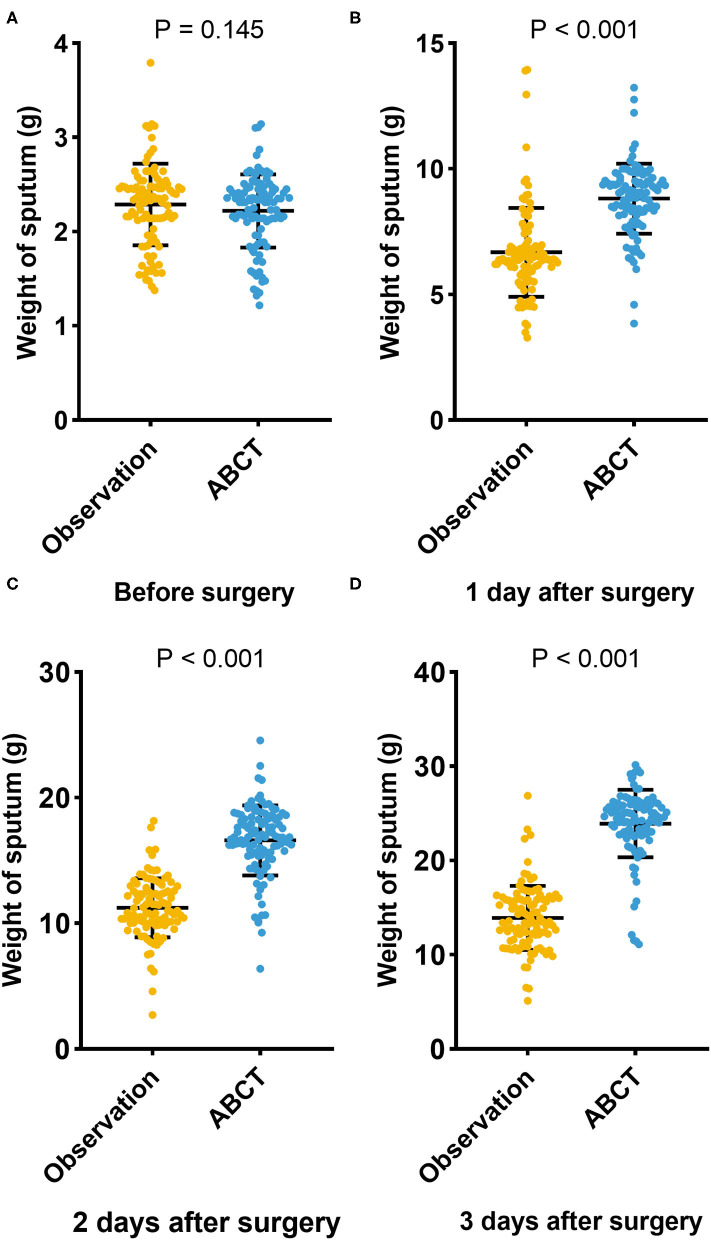
The weight of sputum in two groups before surgery **(A)**, in 1 day after surgery **(B)**, in 2 days after surgery **(C)**, in 3 days after surgery **(D)**.

**Figure 4:**
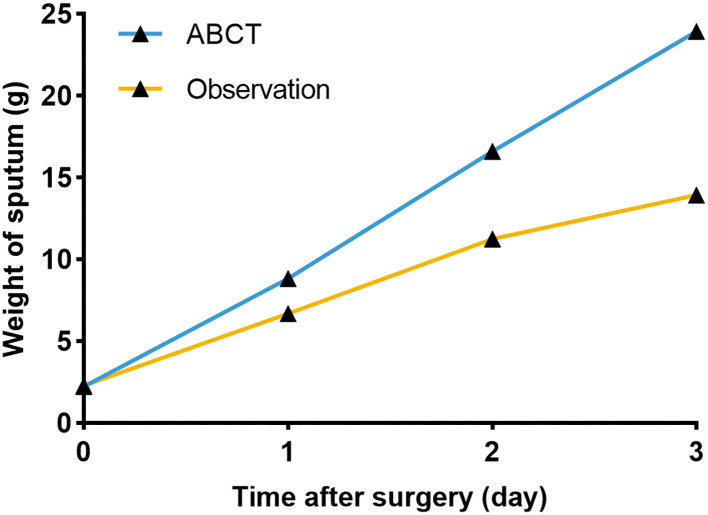
The line chart of comparing the weight of sputum between the two groups.

We collected the sputum of the patients before and after surgery (the first, second, and third postoperative day). Before surgery, the mean weight of the sputum was 2.255 ± 0.412 g, and no significant differences were observed in both groups (*P* = 0.206; Figure 3A). The mean weight of the sputum collected on the first, second, and third day after surgery was shown in Table 1. We found that the weight of the sputum in the ACBT group was heavier than that in the observation group, and a significant difference was observed between the two groups (Figures 3, 4; all *P* < 0.05).

**Figure 5:**
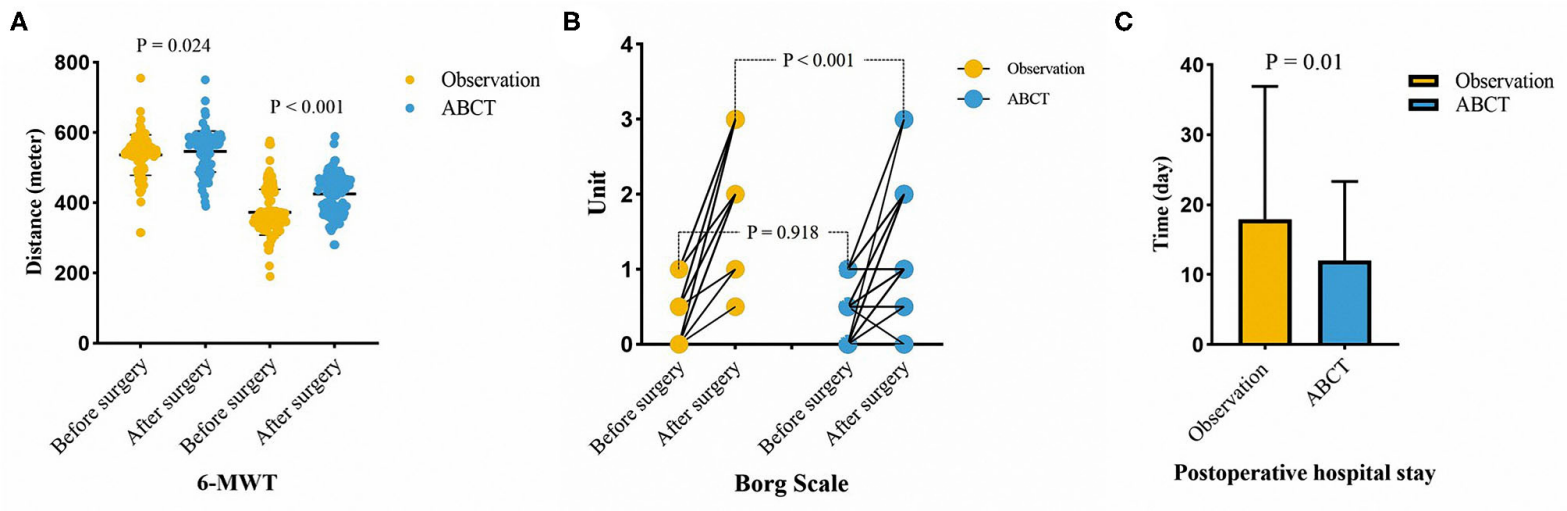
The results of 6-MWT **(A)**, Borg scale **(B)**, and the time of postoperative hospital stay **(C)** in two groups.

To reduce confounding factors in both groups, we required the patients to undergo the 6-MWT before surgery. Ten days after surgery, the patients were instructed to undergo the 6-MWT again. The outcomes were better in the ACBT group than in the observation group both in preoperative and postoperative period (Table 1 and Figure 5A).

The Borg scale was used to assess the tolerance grade of patients who engaged in aerobic exercises. In this study, the 6-MWT was utilized as an aerobic exercise. When the patients finished the 6-MWT after surgery, professional nurses helped them evaluate themselves using the Borg scale. Before surgery, the Borg scale scores of the two groups didn't differ; however, after surgery, the ACBT group had a lower grade than had the observation group (Table 1 and Figure 5B). A lower grade indicated better tolerance in patients who engaged in aerobic exercises.

### Anastomotic Leakage and Hospital Stay

The ACBT group had a lower incidence rate of anastomotic leakage than the observation group (Table 1, *P* = 0.001). The mean length of hospital stay, which was the focus of doctors and patients, was 17.953 ± 18.961 days in the observation group and 12.037 ± 11.321 days in the ACBT group. We also found that the observational group had a higher discharge ratio over 2 weeks in all cohorts (adjusted OR 2.487, P = 0.021, Table 3). Thus, ACBT training could decrease the length of hospital stay (Table 1 and Figure 5C; *P* = 0.01).

**Table 3:** Multivariable Logistic regression analysis for length of hospital stay according to different groups in all patients.

	**Discharge over 2 weeks**		
	***OR***	**95% CI**	***P*-Value**
**Group**(ACBT vs. observation)	2.487	1.147-5.392	0.021
**Age** (year)	1.047	1.000-1.096	0.05
**Anastomotic leakage**			
(No vs. yes)	2.618	0.762-8.995	0.126
**Open surgery**			
(No vs. yes)	5.564	2.220-13.942	<0.001
**Surgical approach**			<0.001
McKeown	1	reference	
Ivor-Lewis	1.323	0.250-6.996	0.742
Sweet	9.933	1.803-54.726	0.008

*ACBT, active cycle of breathing technique; OR, odd ratio; CI, confidence interval; method was “Enter selection”*.

## Discussion

To the best of our knowledge, this study is the first to observe ACBT training in EC patients. The cases were divided into the observation and ACBT groups. We provided ACBT training before surgery, with the hope that this technique could improve the outcomes of rapid rehabilitation in patients after esophagectomy. In the course of the study, we used the 6-MWT and Borg scale to evaluate the tolerance of aerobic exercise in patients with EC before and after surgery. We collected data about weight of sputum, smoking index, tumor location, pTNM stage, pathway of surgery, surgical approach, anastomotic leakage, and length of postoperative hospital stay. These data were analyzed using statistical tools. The results of this study confirmed that ACBT could increase the weight of sputum and tolerance to aerobic exercises after surgery in EC patients, based on the results of Yang Mei's study about lung cancer (25). To reduce confounding effect of the smoking index, age, surgical approaches, and tumor location on the observed indicators, we used the chi-squared test, CMH test, and Kruskal–Wallis test to assess the data before surgery. We found that the mean weight of sputum in the observation group was lighter than that in the ACBT group. On the basis of the above-mentioned results, ACBT training could increase the weight of sputum after esophagectomy; thus, patients should receive ACBT training before surgery to help them cough sputum much easier. Using the Borg scale, we found that the mean score was not different between the observation and ACBT groups before surgery; however, the 6-MWT scores before surgery differed between the observation and ACBT groups. After surgery, the ACBT group had better outcomes than the observation group. Based on the analysis of pre-surgery data, we found that the mean distance achieved in the 6-MWT was longer in the ACBT group than in the observation group (*P* = 0.024). We combined the scores of the 6-MWT and Borg scale to assess tolerance of aerobic exercise, and results showed that ACBT could improve this tolerance after surgery.

We paid more attention to the findings associated with complications, which were a major concern of our research. Anastomotic leakage was considered to be the main cause of prolonged hospitalization. In this research, 16 patients had anastomotic leakage, among which 1 case occurred in the ACBT group and 15 cases occurred in the observation group. The occurrence rate of anastomotic leakage was significantly different between the two groups. We speculated that ACBT might decrease the occurrence of anastomotic leakage.

The length of postoperative hospital stay decreases with the reduction in complication rate. In our study, the ACBT group had a shorter length of postoperative hospital stay than the observation group. Thus, ACBT was likely to improve the outcomes of postoperative surgery in EC patients. Compared with previous studies, this study had a larger sample size and a higher number of patients who underwent esophagectomy.

The present study had some limitations. It was conducted at a single institution, and the sample size was relatively small. Therefore, multicenter studies with larger cohorts must be conducted to validate the results of the current study. Lacking related information of BMI, nutrition status and neo-adjuvant therapy was another drawback. In future studies, these factors should be taken into consideration to evaluate their impact on perioperative outcomes. Moreover, although patients with various histological types were included in this study, squamous cell carcinoma took the lead due to its prevalence in China and thus causing the disproportion of histology type. Given that it was the perioperative outcomes that we focused on, unbalance in the histology type barely had impact on the results. Nevertheless, these patients required longer observation and follow-up. Thus, prospective studies must be performed to validate the results of this study.

## Conclusion

In a conclusion, ACBT is likely to help patients of EC improve perioperative outcome after esophagectomy, which may decrease the time of hospital stay. However, more prospective studies are needed to explore the impact of ACBT on postoperative complications in EC patients after esophagectomy.

## Data Availability Statement

The datasets presented in this study can be found in online repositories. The names of the repository/repositories and accession number(s) can be found below: https://www.researchdata.org.cn/Default.aspx. Approval number: RDDA2019001345.

## Ethics Statement

The studies involving human participants were reviewed and approved by Ethics Committee of Sun Yat-sen University Cancer Center. The patients/participants provided their written informed consent to participate in this study.

## Author Contributions

C-ZL, G-WM, J-DZ, and HY designed the research. L-LW and S-WZ processed data and wrote the draft. S-WZ, MW, C-ZL, and W-JW recorded the data of patients. All authors observed the patients. HY, G-WM, W-JW, and J-DZ reviewed and edited the article. All authors contributed to the article and approved the submitted version.

## Funding

This work was supported by Guangdong Esophageal Cancer Institute Science and Technology Program (No. Q201601).

## Conflict of Interest

The authors declare that the research was conducted in the absence of any commercial or financial relationships that could be construed as a potential conflict of interest.

## Publisher's Note

All claims expressed in this article are solely those of the authors and do not necessarily represent those of their affiliated organizations, or those of the publisher, the editors and the reviewers. Any product that may be evaluated in this article, or claim that may be made by its manufacturer, is not guaranteed or endorsed by the publisher.
